# Different Light Wavelengths Differentially Influence the Progression of the Hypersensitive Response Induced by Pathogen Infection in Tobacco

**DOI:** 10.3390/antiox14080954

**Published:** 2025-08-03

**Authors:** Bao Quoc Tran, Anh Trung Nguyen, Sunyo Jung

**Affiliations:** 1 BK21 FOUR KNU Creative BioResearch Group, School of Life Science and Biotechnology, Kyungpook National University, Daegu 41566, Republic of Korea; baotran509@gmail.com (B.Q.T.); nguyenanh18190@knu.ac.kr (A.T.N.); 2 Institute of Life Science and Biotechnology, College of Natural Sciences, Kyungpook National University, Daegu 41566, Republic of Korea

**Keywords:** chlorophyll metabolism, hypersensitive response, oxidative stress, *Pseudomonas syringae*, ROS detoxification, tobacco

## Abstract

Using light-emitting diodes (LEDs), we examined how different light wavelengths influence the hypersensitive response (HR) in tobacco plants infected with *Pseudomonas syringae* pv. *tomato* (Pst). Pst-infiltrated plants exhibited greater resistance to Pst infection under green and blue light compared to white and red light, as indicated by reduced HR-associated programmed cell death, lower H_2_O_2_ production, and up to 64% reduction in membrane damage. During the late stage of HR, catalase and ascorbate peroxidase activities peaked under green and blue LEDs, with 5- and 10-fold increases, respectively, while superoxide dismutase activity was higher under white and red LEDs. Defense-related genes *CHS1*, *PALa*, *PR1*, and *PR2* were more strongly induced by white and red light. The plants treated with green or blue LEDs during Pst infection prompted faster degradation of phototoxic Mg-porphyrins and exhibited smaller declines in *F*_v_/*F*_m_, electron transport rate, chlorophyll content, and *LHCB* expression compared to those treated with white or red LEDs. By contrast, the induction of the chlorophyll catabolic gene *SGR* was 54% and 77% lower in green and blue LEDs, respectively, compared to white LEDs. This study demonstrates that light quality differentially affects Pst-mediated HR, with green and blue light more effectively suppressing HR progression, mainly by reducing oxidative stress through enhanced antioxidative capacity and mitigation of photosynthetic impairments.

## 1. Introduction

Biotic stress results in the overaccumulation of reactive oxygen species (ROS) and triggers defense responses, including the expression of phenyl ammonia lyase (PAL), chalcone synthase (CHS), and pathogenesis-related (PR) proteins [1,2,3]. Infection of plants by *Pseudomonas syringae* can rapidly activate programmed cell death (PCD) in association with disease resistance [4]. Chloroplasts play a crucial role as sources of signals that trigger plant PCD [5,6,7], and damage to the chloroplast membrane can lead to ROS accumulation [5,6]. In the presence of light, chloroplasts are the primary source of ROS, whereas mitochondria are considered the main source in non-photosynthetic tissues [7]. Chloroplast-derived molecules involved in the initiation of PCD, chloroplast-to-nucleus retrograde signaling, or stress responses include hydrogen peroxide (H_2_O_2_), singlet oxygen (^1^O_2_), and Mg-protoporphyrin IX (Mg-Proto IX) [8,9].

Both chlorophyll and light-harvesting chlorophyll-binding proteins (LHCs) are essential structural components in the photosynthetic apparatus [10,11]. Chlorophyll biosynthesis shares early biosynthetic steps with other porphyrins such as heme and siroheme in plants [12,13]. After the conversion of 5-aminolevulinic acid into Proto IX, an Mg^2+^ ion is inserted into Proto IX to produce Mg-Proto IX, the first intermediate in the chlorophyll branch of the porphyrin biosynthetic pathway, namely the Mg branch. The biosynthesis and degradation of porphyrins are highly regulated to prevent the accumulation of photoreactive porphyrin intermediates that can undergo photooxidation, resulting in the production of ROS and subsequent promotion of cell death [5,14]. The accumulation of protochlorophyllide (Pchlide) in *Arabidopsis flu* mutants [15] results in cell death phenotypes.

The absorption of excess light energy leads to the generation of ROS within photosynthetic machinery [16]. The conversion of light energy into chemical energy during photosynthesis results in a reduction in chlorophyll *a* fluorescence, a process known as photochemical quenching (q_P_) [17]. Excess ROS are scavenged by ROS-detoxifying enzymes [18,19]. In plants, the primary enzyme responsible for ROS detoxification is superoxide dismutase (SOD), which transforms superoxide (O_2_^−^) into H_2_O_2_, thus protecting cells from oxidative stress [20,21]. Catalase (CAT) and ascorbate peroxidase (APX) catalyze the elimination of the toxic product of SOD detoxification [16,22]. High ROS concentrations can alter cells’ antioxidant status, leading to cell death [7].

Light wavelengths are a key environmental factor regulating plant growth and morphogenesis, as demonstrated by numerous studies [23,24,25]. However, the impact of different light spectra on the progression of pathogen-mediated hypersensitive response (HR) remains unexplored. We examined HR, oxidative stress, and photosynthetic function in tobacco plants infected with *P.* *syringae* pv. *tomato* (Pst) under various light wavelengths. We analyzed changes in ROS detoxification activity and gene expression patterns associated with defense mechanisms during HR progression. Additionally, we assessed how different light wavelengths influence the levels of photosensitizing porphyrin intermediates upon Pst infection.

## 2. Materials and Methods

### 2.1. Plant Growth, Light Treatment, and Pathogen Inoculation

Tobacco (*Nicotiana tabacum* var. Samsun NN) plants were grown in a growth chamber set to 24 °C with a 16 h light and 8 h dark cycle and a photosynthetic photon flux density (PPFD) of 300 μmol m^−2^ s^−1^. To produce the bacterial inoculant, *Pseudomonas syringae* pathovar *tomato* strain DC3000 (Pst DC3000) was grown in King’s B medium at 28 °C with continuous agitation. The bacteria culture was centrifuged at 3500× *g*, and the pellet was resuspended in 10 mM MgSO_4_ and adjusted to an OD_600_ of 0.008. Six-week-old tobacco plants were acclimated for two days under one of four different types of light-emitting diodes (LEDs) (Union LED Electronic Co., Siheung-si, Republic of Korea): broad-spectrum white LED (420–680 nm), green LED (520–550 nm), blue LED (460–490 nm), or red LED (620–650 nm). Then, the undersides of fully expanded leaves were inoculated with the suspension via the infiltration method using a needless 1 mL syringe. Each LED treatment was carried out in individually controlled chambers to prevent spectral interference between treatments. The LED array chambers were set to provide a 16 h light and 8 h dark cycle, with a PPFD of 150 μmol m^−2^ s^−1^. Leaf disks from infiltrated areas or whole leaves were collected at specific time points up to 54 h post-infiltration (hpi) for analysis. Specifically, leaf disks collected at 54 hpi indicate the late stage of PCD.

### 2.2. Conductivity Measurement

Leaf disks were excised from the Pst-infiltrated zones at specified time points and placed in 5 mL miliQ water for 10 min at 20 °C under gyratory agitation (50 rpm). Cellular leakage into the bathing solution was subsequently measured by detecting electrolyte leakage with a conductivity meter (Cole-Parmer Instruments, Vernon Hills, IL, USA).

### 2.3. Lipid Peroxidation

To estimate lipid peroxidation, the production of malondialdehyde (MDA) was measured using a modified thiobarbituric acid method [26]. Leaf tissues were ground in a solution containing 0.5% thiobarbituric acid and 20% trichloroacetic acid. After centrifugation, supernatants were heated in a boiling water bath for 25 min, recentrifuged, and utilized for spectrophotometric analysis at 532 nm.

### 2.4. In Vivo Detection of H_2_O_2_

For the visual detection of H_2_O_2_ in leaves, 3,3-diaminobenzidine (DAB) staining was used [27]. The leaves were excised and incubated in a 1 mg mL^−1^ DAB solution (pH 3.8) for 4 h under light at 25 °C. Subsequently, the reaction was stopped by boiling the leaves in ethanol for 10 min. This treatment decolorizes leaves, leaving only the deep brown polymerization product of the reaction between DAB and H_2_O_2_.

### 2.5. Measurement of Antioxidant Enzyme Activities

Total soluble proteins were extracted by homogenizing leaf tissues in 0.1 M potassium phosphate buffer (pH 7.5) containing 1% PVP-40, 1 mM phenylmethylsulfonyl fluoride, and 2 mM EDTA, followed by centrifugation at 15,000× *g* for 20 min at 4 °C. SOD activity was spectrophotometrically measured by the xanthine oxidase-cytochrome *c* method [28]. The assay was performed at 25 °C in a 3-mL volume containing 50 mM sodium carbonate buffer (pH 10.0), 0.05 mM xanthine, 0.1 mM EDTA, and 15 µM ferricytochrome *c* by adding xanthine oxidase. Enzyme activity (units/mg protein) is proportional to (V/v − 1)/mg protein, where V and v represent the change in absorbance (550 nm) per min in the absence and presence of SOD, respectively. An assay for APX activity was performed spectrophotometrically [29]. The 3 mL reaction volume contained 0.1 M potassium phosphate buffer (pH 7.5), 0.2 mM H_2_O_2_, and 0.5 mM ascorbate at 25 °C. The reaction was initiated with the addition of H_2_O_2_. APX activity was performed by measuring the decrease in absorbance of ascorbate at 290 nm at 25 °C as ascorbate was oxidized. CAT activity was measured spectrophotometrically in a 3 mL volume containing 50 mM potassium phosphate buffer (pH 7.0) and 20 mM H_2_O_2_ by monitoring the destruction of H_2_O_2_ at 240 nm [30].

### 2.6. RNA Extraction and RT-qPCR Analysis

Total RNA was extracted from leaf tissues using TRIzol Reagent (Invitrogen, Carlsbad, CA, USA) in accordance with the manufacturer’s instructions. Five µg of RNA from each sample was utilized to synthesize cDNA via the reverse transcription reaction (*ImProm-II™* Reverse Transcription System, Promega, Madison, WI, USA). Subsequently, qPCR analyses were performed with a StepOnePlus™ Real-Time PCR system (Applied Biosystems, Waltham, MA, USA) using cDNA from each sample, Power SYBR™ Green PCR Master Mix (Applied Biosystems), and gene-specific primers (Supplementary Table S1). The RT-qPCR program consisted of 2 min at 50 °C and 10 min at 95 °C, and then 40 cycles of 15 s at 95 °C and 1 min at 60 °C. The β-*Tubulin* gene was used as an internal control. For analysis, the white LEDs–0 hpi sample served as a calibrator, with its expression level normalized to 1.

### 2.7. Chlorophyll a Fluorescence Measurement

Chlorophyll *a* fluorescence parameters were assessed with a pulse amplitude modulation fluorometer (JUNIOR-PAM, Walz, Effeltrich, Germany). Plants from each treatment were kept in darkness for 30 min to adapt. The minimum fluorescence (*F*_o_) was recorded using a weak measuring beam emitted by a pulse light-emitting diode, while the maximum fluorescence (*F*_m_) was determined after exposing the leaf surface to a saturating pulse of white light. The *F*_v_/*F*_m_ value, indicating photosystem II (PSII) activity, was utilized to evaluate functional damage in the plants. The electron transport rate (ETR) is calculated based on the photochemical quantum yield of PSII. The q_P_ value, used to calculate photochemical quenching, was characterized as q_P_ = (*F*_m_′ − *F*′)/(*F*_m_′ − *F*_o_′) [31].

### 2.8. Determination of Porphyrin Contents

Chlorophyll content was measured using a spectrophotometer following the procedure by Lichtenthaler [32]. For measurement of porphyrin contents, leaf tissue was homogenized in a mixture of methanol/acetone/0.1 N NaOH (9/10/1) and then centrifuged at 10,000× *g* and 4 °C [33]. Porphyrin intermediates were analyzed using high-performance liquid chromatography (HPLC) with a Novapak C_18_ column (4 µm particle size, 4.6 × 250 mm, Waters, Milford, MA, USA) at a flow rate of 1 mL min^−1^. A gradient solvent system was initiated with 0.1 M ammonium phosphate (pH 5.8) and methanol (20:80, *v*/*v*) for 10 min, followed by a linear gradient to 100% methanol over 8 min, which was then maintained for 17 min. The eluate was monitored using a fluorescence detector (2474, Waters) at excitation and emission wavelengths of 440 and 630 nm for protochlorophyllide (Pchlide), and 415 and 595 nm for MgProto IX and MgProto IX methyl ester (MgProto ME), respectively [34].

### 2.9. Statistical Analysis

All data are shown as means ± standard error (SE). Statistically significant differences were determined by Duncan’s multiple range test (at *p* < 0.05) using SPSS 27 software (SPSS Inc., Chicago, IL, USA).

## 3. Results

### 3.1. The Impact of Different Light Wavelengths on HR Induced by P. syringae Infection

**Figure 1:**
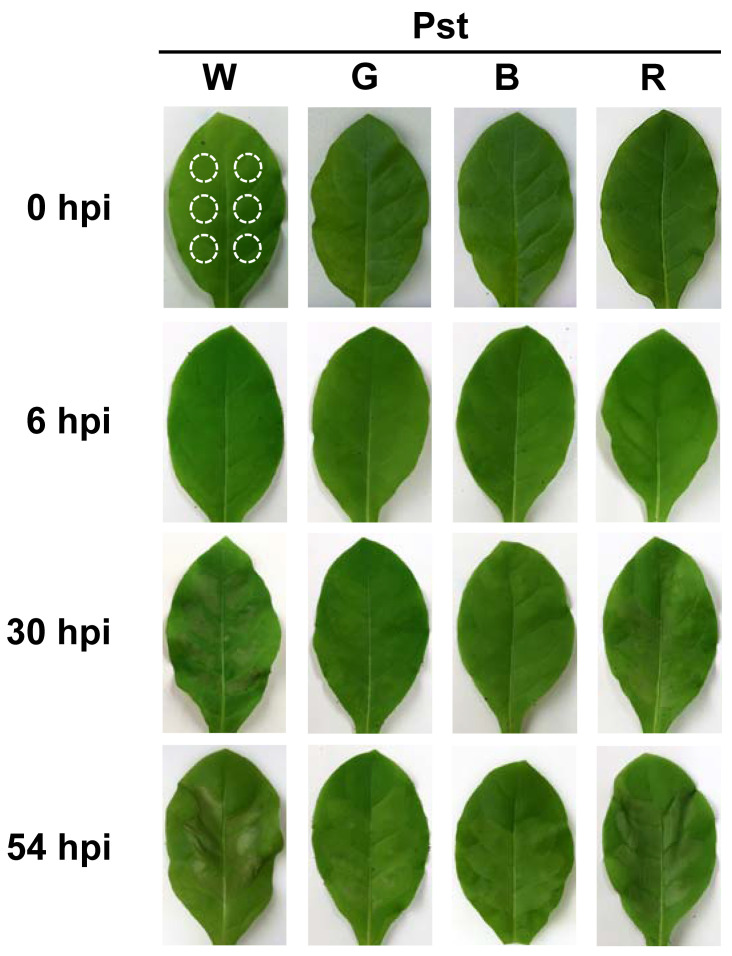
Effects of different light wavelengths on HR in tobacco leaves infected with *P. syringae* pv. *tomato* (Pst). Leaves of 6-week-old tobacco plants were inoculated with Pst and exposed to different light-emitting diodes (LEDs). The treatments included control plants (uninoculated at 0 hpi) and Pst-inoculated plants under broad spectrum white (W; 420–680 nm), green (G; 520–550 nm), blue (B; 460–490 nm), and red (R; 620–650 nm) LEDs. Images of leaves were taken at different time points following Pst inoculation (hpi, hours post-infiltration). The dotted circles indicate the sites of Pst infiltration.

The effects of different light qualities on HR triggered by *P. syringae* infection were examined using LEDs of different wavelengths. Six-week-old tobacco plants were inoculated with Pst to induce HR and exposed to white, green, blue, or red LED light. At 30 hpi, leaf areas infected by Pst started exhibiting mild necrosis, i.e., HR, under the white and red LED treatments, which progressed to severe necrosis by 54 hpi (Figure 1). Plants under green and blue LEDs did not exhibit leaf necrosis at 30 hpi but developed noticeable necrosis by 54 hpi. During Pst infection, plants exposed to white LEDs exhibited the most severe necrosis, followed by those under red, green, and blue LED treatments, in that order.

**Figure 2:**
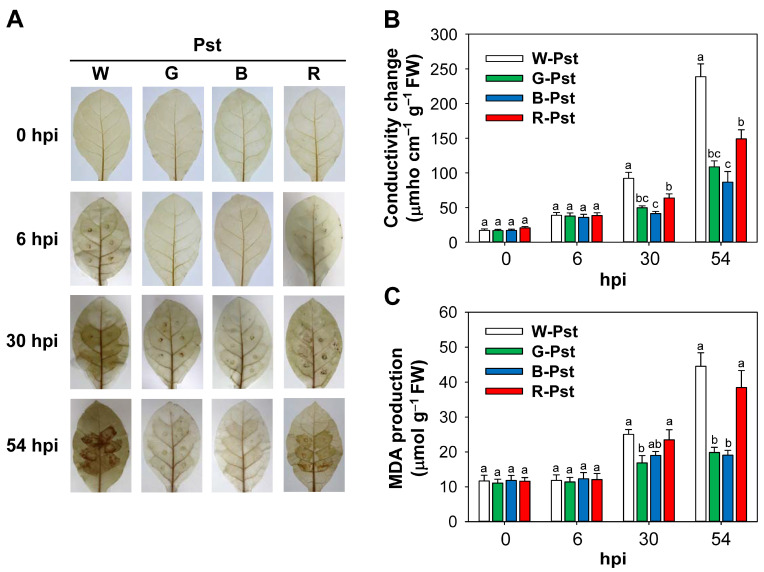
Effects of different light wavelengths on oxidative stress and membrane integrity in leaves infected with Pst. (**A**) DAB staining of H_2_O_2_ in leaves. The brown spots indicate H_2_O_2_ production. (**B**) Conductivity change. (**C**) MDA accumulation. Plants were exposed to the treatments described in the caption of Figure 1. W-Pst, G-Pst, B-Pst, and R-Pst represent plants inoculated with Pst under white, green, blue, and red LEDs, respectively. Data are expressed as means ± SE of nine replicates from three independent experiments. Means with the same letter are not significantly different at *p* < 0.05 according to Duncan’s Multiple Range Test.

To determine whether different light wavelengths influence ROS levels during HR, untreated control and Pst-infected leaves were incubated with DAB to detect H_2_O_2_ generation. Pst-infected leaves began to exhibit faint brown spots as early as 6 hpi under the white and red LEDs, and the spots became stronger by 30 and 54 hpi (Figure 2A). However, under green and blue LEDs, brown spots were not noticeable in Pst-infected leaf areas at 6 hpi but became visible at 30 and 54 hpi. The more pronounced spots observed under white and red LEDs indicated that Pst infection led to significantly higher H_2_O_2_ production under these conditions.

Conductivity, a marker for cellular leakage, was assessed during HR development under the different LED treatments. It continuously increased in Pst-infected leaves from 6 to 54 hpi under all LED treatments (Figure 2B), indicating impaired membrane integrity. Plants exposed to white or red LEDs exhibited a greater increase in conductivity to Pst infection compared to those under blue or green LEDs, with the highest increase in white LED treatment, reaching a 14-fold increase. To further verify membrane integrity, we evaluated MDA content, an indicator of lipid peroxidation. The MDA levels began to increase in response to Pst infiltration under all LED treatments at 30 hpi, with greater increases under white and red LEDs than green and blue LEDs (Figure 2C). At 54 hpi, white and red LED treatments resulted in MDA levels that were up to twice those observed with green and blue LED treatments, confirming greater membrane damage under white and red LEDs.

### 3.2. The Impact of Light Quality on the Regulation of ROS Detoxification and Defense Responses During Pst-Induced HR

**Figure 3:**
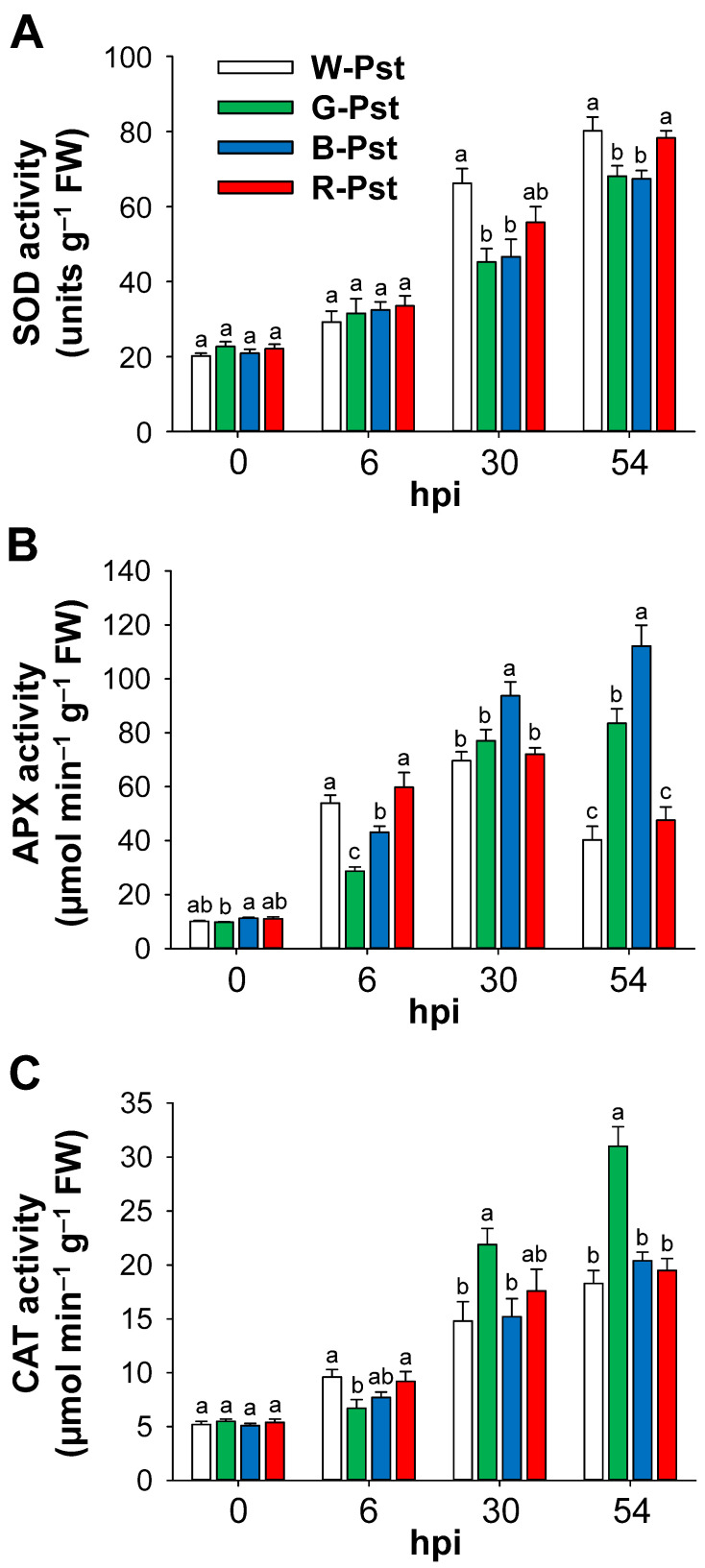
Effects of different light wavelengths on the activity of ROS-detoxifying enzymes in leaves infected with Pst. (**A**) SOD. (**B**) APX. (**C**) CAT. Data are expressed as means ± SE of nine replicates from three independent experiments. Plants were exposed to the treatments described in the caption of Figure 1. The treatment notations are the same as those in Figure 2. Means with the same letter are not significantly different at *p* < 0.05 according to Duncan’s Multiple Range Test.

Following Pst infection, we monitored how LED lights of different wavelengths influenced the regulation of major ROS-detoxifying enzymes and defense-related genes. In response to Pst infection, the activities of SOD increased under all LED treatments at 6 hpi compared to the untreated control (Figure 3A). At 30 and 54 hpi, SOD activity continued to increase, but the increase was greater under white and red LEDs than under green and blue LEDs. APX activity markedly increased at 6 hpi under white and red LED treatments but decreased by 54 hpi, although it remained higher than that of the control (Figure 3B). Under green and blue LEDs, the activities of APX increased gradually over 54 h. Plants exposed to blue LEDs showed the highest APX activity among all LED treatments at 30 and 54 hpi. During Pst infection, the activities of CAT steadily increased until 54 hpi in all LED treatments (Figure 3C). At 30 and 54 hpi, CAT activity was higher under green LEDs compared to other LEDs, with the highest activity observed at 54 hpi.

**Figure 4:**
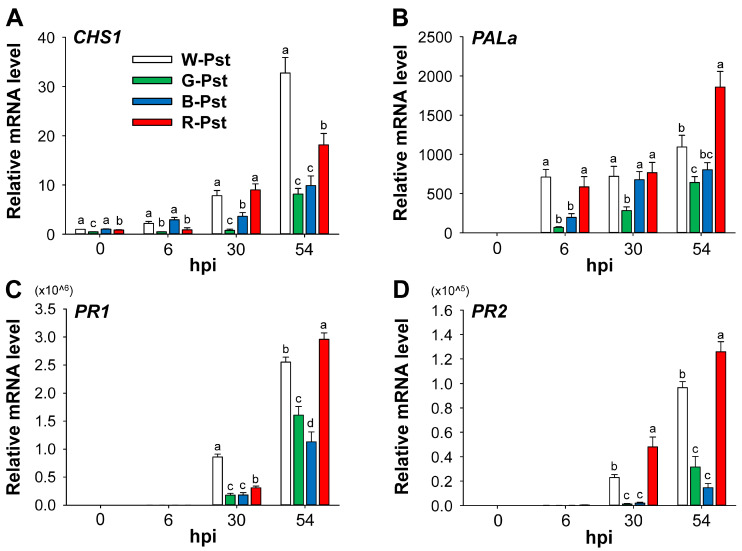
Effects of different light wavelengths on the expression profile of defense-involved genes in leaves infected with Pst. (**A**) *CHS1*. (**B**) *PALa*. (**C**) *PR1*. (**D**) *PR2*. β-*Tubulin* was used as an internal control, and the white LEDs–0 hpi sample was used for normalization, with its expression level set to 1. Plants were exposed to the treatments described in the caption of Figure 1. The treatment notations are the same as those in Figure 2. Data are expressed as means ± SE of nine replicates from three independent experiments. Means with the same letter are not significantly different at *p* < 0.05 according to Duncan’s Multiple Range Test.

The expression of *CHS1*, a key component in plant defense, was higher under white and blue LEDs at 6 hpi compared to the control and was noticeably upregulated under white and red LEDs at 30 hpi (Figure 4A). By 54 hpi, the expression continued to increase, with greater upregulation under white and red LEDs than under green and blue LEDs. Within 6 h of Pst infection, at which time no necrosis was visible, *PALa* was dramatically upregulated under all LED treatments, with the greatest increase under red LEDs at 54 hpi (Figure 4B). The *PR1* and *PR2* genes were noticeably upregulated in response to Pst infection at 30 hpi, with their expression further increasing by 54 hpi under all LED treatments (Figure 4C,D). However, the increase was more pronounced under white and red LEDs compared to green and blue LEDs (Figure 4). These results show that Pst-induced HR leads to light quality-dependent activation of ROS detoxification and defense responses under different light wavelengths.

### 3.3. The Impact of Light Quality on Regulating Porphyrin Levels During Pst-Induced HR

**Figure 5:**
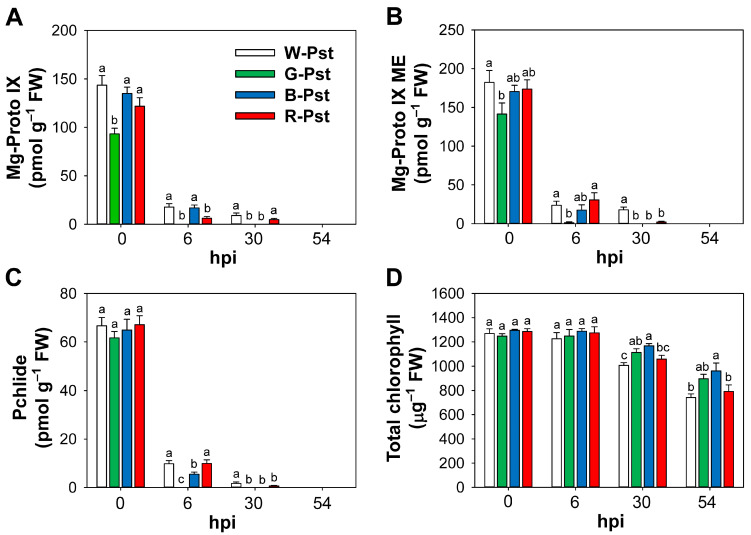
Effects of different light wavelengths on metabolites in the chlorophyll branch of the porphyrin biosynthetic pathway in leaves infected with Pst. (**A**–**C**) Mg-porphyrin intermediates. (**D**) Total chlorophyll. Plants were exposed to the treatments described in the caption of Figure 1. The treatment notations are the same as those in Figure 2. Data are expressed as means ± SE of nine replicates from three independent experiments. Means with the same letter are not significantly different at *p* < 0.05 according to Duncan’s Multiple Range Test.

To assess the effect of light quality on intermediates in the chlorophyll branch of the porphyrin pathway during Pst infection, we examined the porphyrin status in Pst-infected plants subjected to various LED light wavelengths. Mg-Proto IX levels greatly decreased at 6 hpi under white, blue, and red LEDs; Mg-Proto IX was not detected under green LEDs (Figure 5A). Mg-Proto IX methyl ester (ME) levels were also markedly decreased under all LED treatments at 6 hpi, with negligible amounts detected under green LEDs (Figure 5B). By 30 hpi, both Mg-Proto IX and Mg-Proto IX ME further decreased in white and red LED treatments and were undetectable in green and blue LED treatments. Pchlide levels exhibited a decreasing trend similar to that of Mg-Proto IX ME (Figure 5C). By 54 hpi, Mg-Proto IX, Mg-Proto IX ME, and Pchlide were no longer observed under any of the LED treatments.

The levels of chlorophyll, the final product of the chlorophyll branch, were not significantly different among various LEDs at 6 hpi (Figure 5D). However, by 30 hpi, chlorophyll levels decreased by 21%, 18%, 11%, and 10% under white, red, green, and blue LEDs, respectively, compared to the control. By 54 hpi, these decreases reached 42%, 38%, 28%, and 26%, showing greater reductions under white and red LEDs. During Pst-mediated HR, blue and green LEDs induced faster declines in Mg-porphyrin intermediate levels but sustained chlorophyll levels more effectively than white and red LEDs.

### 3.4. The Impact of Light Quality on Photosynthetic Systems During Pst-Induced HR

**Figure 6:**
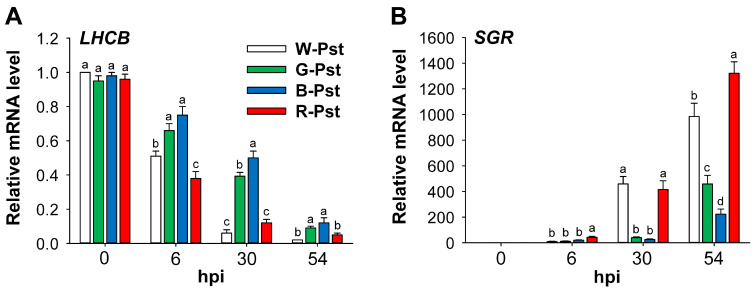
Effects of different light wavelengths on the integrity of the photosystem in leaves infected with Pst. The expression of *LHCB* (**A**) and *SGR* (**B**) genes. β-*Tubulin* was used as an internal control, and the white LEDs–0 hpi sample was used for normalization, with its expression level set to 1. Plants were exposed to the treatments described in the caption of Figure 1. The treatment notations are the same as those in Figure 2. Data are means ± SE of nine replicates from three independent experiments. Means with the same letter are not significantly different at *p* < 0.05 by Duncan’s Multiple Range Test.

We assessed the organization of the photosynthetic machinery in Pst-infected leaves through the quantitative analysis of *LHCB*. Expression levels of *LHCB*, a marker of photosynthetic gene expression patterns, began decreasing at 6 hpi in all LED treatments and continued to decrease at 30 and 54 hpi, with greater reductions under white and red LEDs (Figure 6A). Conversely, the expression of the stay-green gene (*SGR*), an indicator of chlorophyll degradation, increased continuously from 6 to 54 hpi in all LED treatments, with greater increases under white and red LEDs at 30 and 54 hpi (Figure 6B).

The effects of different light wavelengths on photosynthetic performance were also examined using the *F*_v_/*F*_m_ value, a measure of the photochemical quantum efficiency of PSII, during Pst-mediated HR. After Pst infection, *F*_v_/*F*_m_ in plants treated with white or red LEDs continuously decreased from 6 to 54 hpi, with declines of 52% and 34% induced by white and red LEDs, respectively, at 54 hpi (Table 1). Under green LEDs, *F*_v_/*F*_m_ remained constant until 30 hpi and then decreased by 10% at 54 hpi compared to the controls, while under blue LEDs, it showed an 8% decline at 54 hpi. The ETR began to decrease in all LED treatments at 6 hpi, continuing to decrease at 30 and 54 hpi, with the greatest reduction observed under white LEDs, followed by red, green, and blue LEDs in decreasing order (Table 1). The q_P_ values were used to estimate the initiation of photoinhibition in PSII. The q_P_ started to decline at 6 dpi and continued to decrease at 30 and 54 hpi, with the magnitude of declines in the order of white, red, green, and blue LEDs (Table 1). These results indicate that different light wavelengths caused differential impairments in chloroplast function in response to Pst infection.

## 4. Discussion

The HR of plants to pathogen infection appears to involve the induction of PCD and other defense responses [35], but little is known about the effects of light wavelength on the mechanisms underlying pathogen-induced HR. Our study demonstrates that different light wavelengths significantly impact HR-associated PCD, primarily by differentially regulating chloroplast-related events during Pst infection, emphasizing the critical role of the chloroplasts in plant HR. Importantly, in tobacco plants infected with Pst, white and red LED treatments triggered more marked necrosis at 30 and 54 hpi compared to blue and green LED treatments (Figure 1). Supporting this observation, irradiation with blue [36], green [37], or red light [36,38], compared to white light, has been shown to suppress disease symptoms in plants infected with pathogens. The suppression of the HR under blue and green LEDs points out the impact of these wavelengths in delaying the progression of PCD in plants.

Some early events associated with HR relate to the rapid generation of ROS in the form of H_2_O_2_ [39,40]. In Pst-infected leaves, treatments with white or red LEDs led to early H_2_O_2_ detection at 6 hpi, followed by a marked increase by 30 hpi, with the highest H_2_O_2_ levels under white LEDs, whereas lower H_2_O_2_ was formed under green and blue LEDs (Figure 2). The greater production of H_2_O_2_ under white and red LEDs corresponded to marked necrosis occurring at 54 hpi (Figure 1 and Figure 2). White and red LED treatments also significantly increased conductivity compared to blue and green LEDs, with the highest increase under white LEDs (Figure 2). Lipid peroxidation is known to be linked to HR cell death [41]. At 54 hpi, white and red LEDs produced MDA levels that were twice as high as those seen under green and blue LEDs. These results indicate that green and blue light mitigate lipid peroxidation and membrane disruption in the late stage of the HR. In a previous study, levels of MDA and conductivity decreased under red light but increased under blue light compared to white light in *Camptotheca acuminata* [25]; however, this study did not involve pathogen treatment. During Pst infection, lower H_2_O_2_ levels and reduced membrane damage under green and blue LEDs indicate a wavelength-dependent impact on oxidative stress and membrane integrity, potentially contributing to the suppression of HR.

In plant cells, ROS detoxification under oxidative stress relies on the efficiency of enzymatic antioxidant components [18,19]. Although numerous studies have explored antioxidant activities under different light wavelengths [24,25,42,43], the impact of light wavelength on antioxidant responses during pathogen infections has been rarely examined. In the late stage of HR, white and red LEDs enhanced SOD activity more than green and blue LEDs (Figure 3). Previous studies without pathogen treatment showed that SOD activity increased under blue and red light compared to white light [42,44]. At 54 hpi, blue LEDs triggered the highest activity of APX, which primarily scavenges harmful H_2_O_2_ in the chloroplast [21], while CAT activity was highest under green LEDs among all treatments (Figure 3). Green and blue light may help delay HR by lowering H_2_O_2_ levels through the efficient activation of H_2_O_2_-detoxifying enzymes during the late stage of the HR. This wavelength-specific impact is consistent with a previous study in which blue light produced higher *APX1* expression than red and white light in broccoli florets [43]. Similarly, blue LED radiation has been shown to enhance APX activity compared to white light in plants infected with *Botrytis cinerea* [45], and plants overexpressing *APX* exhibited reduced visible HR symptoms [35].

Redox signals, such as H_2_O_2_ accumulation, play a key role in activating plant defenses during the initial phases of HR [46]. CHS and PAL are known to be activated in response to various stress conditions, including fungal or bacterial infection and UV light [2,3]. Under Pst infection, expression of *CHS1* and *PALa* continuously increased under all LED treatments, with notably higher increases under white and red LEDs at 54 hpi (Figure 4). Pathogen-responsive genes *PR-1* and *PR-2* [1,47] were noticeably upregulated at 30 hpi, with even higher levels under white and red LEDs by 54 hpi. Notably, red LEDs induced higher *PALa*, *PR1*, and *PR2* expression than white LEDs at 54 hpi. This result is in accordance with previous observations in *Pseudomonas cichorii*-infected tomato, where *PAL* and *PR-1a* were upregulated more under red light than under white light [48]. Overall, green and blue light led to lower induction of defense-related genes, accompanied by reduced H_2_O_2_ production (Figure 2 and Figure 4), likely due to the alleviation of HR compared to white and red light.

Porphyrin intermediates may act as an endogenous cell death trigger through the production of ^1^O_2_ [9,35]. The chlorophyll branch of the porphyrin pathway begins with the formation of Mg-Proto IX by Mg-chelatase [10,12]. Levels of potent photosensitizers, including Mg-Proto IX, Mg-Proto IX ME, and Pchlide, began to decrease rapidly in response to Pst infection, disappearing by 54 hpi under all LED conditions (Figure 5). The fast disappearance of porphyrin intermediates following Pst infection was suggested to result from photodynamic degradation and reduced porphyrin biosynthesis [49]. Similar declines in porphyrin intermediates have also been previously noted under salt stress, drought, and iron deficiency [34,50,51]. In the late stage of HR, faster scavenging of Mg-porphyrins under green and blue LEDs appears to reduce phototoxicity under Pst infection, thereby helping to delay HR. These results demonstrate that the spectral quality of light regulates porphyrin levels during light quality-dependent HR.

Despite significant declines in the Mg-porphyrin levels, the chlorophyll levels exhibited small reductions under all LED treatments during prolonged Pst infection (Figure 5), indicating limited degradation of chlorophyll. Particularly, green and blue LEDs caused smaller declines in chlorophyll levels compared to white and red LEDs, corresponding to less downregulation of *LHCB* under green and blue LEDs at 30 and 54 hpi (Figure 5 and Figure 6). Previous studies without pathogen infection have also shown that chlorophyll content is higher under blue light compared to white light [24,52], and under green light instead of partial red light compared to red-blue light [53]. Disruption of LHCs can lead to the removal of the porphyrin moiety from chlorophyll [11,40]. Our results indicate that green and blue light induced less photodamage to the photosynthetic apparatus, possibly due to a lower photodegradation rate. This is supported by a lower upregulation of *SGR* under green and blue LEDs (Figure 6B), indicating reduced chlorophyll degradation. The SGR protein extracts Mg^2+^ from chlorophyll *a* to synthesize pheophytin *a* [54] and plays a key role in initiating chlorophyll degradation [39].

Chloroplasts generate contrasting signals that can either inhibit or facilitate cell death [55,56]. In Pst-infected plants, the photosynthetic performance of chloroplasts, as indicated by the *F*_v_/*F*_m_ and ETR values, declined more under white or red LEDs compared to green or blue LEDs, with the greatest decline observed under white LEDs (Table 1). This indicates a greater impairment of PSII activity under white and red LEDs, possibly as a result of excessive reduction of the electron transport system. Similar negative impacts of red light on photosynthesis were observed in other plants that were not experiencing pathogen stress [57,58], whereas green light facilitated more efficient photosynthesis than red light [59]. A decline in q_P_ values indicates increased excitation pressure on PSII [60]. At 30 and 54 hpi, the larger declines in both q_P_ and chlorophyll levels under white and red LEDs, compared to green and blue LEDs (Figure 5 and Table 1), suggest that PSII is more extensively damaged by white and red light. In contrast, the relatively small decline in q_P_ observed under blue LEDs indicates a reduced excitation pressure on PSII, allowing for more efficient maintenance of excitation energy for photochemistry. These results show how specific LED wavelengths differentially modulate the integrity of photosystem during Pst infection, highlighting the protective effects of green and blue light, in contrast to the exacerbating effects of white and red light. The observed benefits of green and blue light suggest potential applications of specific light spectra in controlled agricultural environments to reduce pathogen-induced damage by mitigating oxidative stress and preserving photosynthetic efficiency.

## 5. Conclusions

Our findings demonstrate that green and blue light prominently delay Pst-induced HR, as evidenced by diminished necrosis and lower levels of membrane damage and oxidative stress, compared to white and red light. Green and blue LEDs resulted in the highest activities of CAT and APX, respectively, likely contributing to reduced HR by effectively detoxifying H_2_O_2_ during the late stage of Pst infection. As HR progressed, green and blue LEDs caused faster declines in Mg-porphyrin levels than white and red LEDs. This may play a protective role in mitigating photodamage to PSII by reducing photosensitivity, which is partly related to a lower susceptibility to photosynthetic impairment under green and blue LEDs. The suppression of HR under green and blue light may be attributed to the tight regulation of chloroplast-related events and enhanced antioxidant properties, aiding plants in coping with oxidative stress during Pst infection. This study provides new insights into how different light wavelengths influence the progression of HR, making a significant advancement in understanding their impact on plant processes related to PCD. Further studies on other economically important crop species and pathogen systems will clarify the broader applicability of light quality-based HR modulation in crop protection.

## Figures and Tables

**Table 1:** Effects of different light wavelengths on photosynthetic function in leaves infected with Pst. Plants were exposed to the treatments described in the caption of Figure 1. The treatment notations are the same as those in Figure 2. Data are expressed as means ± SE of nine replicates from three independent experiments. Means with the same letter within a column are not significantly different at *p* < 0.05 based on Duncan’s Multiple Range Test.

LED Treatment		*F*v/*F*m	ETR	q_P_
0 hpi	White	0.813 ± 0.007 ^a^	89.3 ± 2.1 ^a^	0.667 ± 0.021 ^a^
	Green	0.809 ± 0.004 ^a^	85.5 ± 3.7 ^a^	0.651 ± 0.013 ^a^
	Blue	0.810 ± 0.004 ^a^	89.2 ± 2.0 ^a^	0.659 ± 0.012 ^a^
	Red	0.811 ± 0.003 ^a^	86.8 ± 2.0 ^a^	0.644 ± 0.014 ^a^
6 hpi	White	0.763 ± 0.017 ^b^	70.9 ± 1.7 ^b^	0.623 ± 0.011 ^a^
	Green	0.801 ± 0.004 ^a^	78.4 ± 1.8 ^a^	0.638 ± 0.016 ^a^
	Blue	0.807 ± 0.003 ^a^	79.4 ± 1.8 ^a^	0.637 ± 0.020 ^a^
	Red	0.779 ± 0.007 ^ab^	70.1 ± 2.7 ^b^	0.619 ± 0.013 ^a^
30 hpi	White	0.678 ± 0.026 ^c^	53.5 ± 3.0 ^c^	0.466 ± 0.021 ^c^
	Green	0.791 ± 0.009 ^a^	66.4 ± 2.3 ^ab^	0.532 ± 0.011 ^ab^
	Blue	0.755 ± 0.011 ^ab^	75.0 ± 3.6 ^a^	0.584 ± 0.024 ^a^
	Red	0.735 ± 0.018 ^b^	59.5 ± 5.4 ^bc^	0.499 ± 0.016 ^bc^
54 hpi	White	0.389 ± 0.039 ^c^	18.5 ± 5.5 ^c^	0.229 ± 0.027 ^d^
	Green	0.730 ± 0.017 ^a^	42.7 ± 3.5 ^ab^	0.399 ± 0.013 ^b^
	Blue	0.749 ± 0.016 ^a^	55.3 ± 3.6 ^a^	0.503 ± 0.030 ^a^
	Red	0.535 ± 0.035 ^b^	31.7 ± 4.3 ^bc^	0.326 ± 0.011 ^c^

## Data Availability

Data will be made available on request.

## Supplementary Materials

The following supporting information can be downloaded at https://www.mdpi.com/article/10.3390/antiox14080954/s1, Table S1: Primer used for RT-qPCR assays.

## Abbreviations

The following abbreviations are used in this manuscript:

APX	Ascorbate peroxidase
CAT	Catalase
hpi	hours post-infiltration
HR	Hypersensitive response
LEDs	Light-emitting diodes
LHC	Light-harvesting chlorophyll-binding proteins
MDA	Malondialdehyde
PCD	Programmed cell death
Proto IX	Protoporphyrin IX
Proto IX ME	Protoporphyrin IX methyl ester
Pchlide	Protochlorophyllide
Pst	*Pseudomonas syringae* pathovar *tomato*
SOD	Superoxide dismutase
