# Supplementary material for: Different Light Wavelengths Differentially Influence the Progression of the Hypersensitive Response Induced by Pathogen Infection in Tobacco
Source: Antioxidants (Basel). 2025 Aug 3;14(8):954. doi: 10.3390/antiox14080954 (PMC12382845; doi:10.3390/antiox14080954)
Supplement: Supplementary file 1 [file antioxidants-14-00954-s001.zip › antioxidants-3775421-supplementary.pdf]

Table S1. Primers used for RT-qPCR assays.

Gene	Gene accession No.	Primer sequence
CHS1	KU949017	F: TCGAACAAAGGCCTCTCGAC
		R: AGATCACCGCTGTCACGTTT
PR1	D90196	F: TAGCTGAGGGAAGTGGCGAT
		R: TCCACACACCTGTCCTTGTG
PR2	M60460	F: TTAGCGAATACCAACCCGCC
		R: ACATTGGCTAAGAGTGGAAGGT
PALa	AB008199	F: AGGTCATCCGTTCTGCAACC
		R: TTGGAGTGCCCTGGAAGTTG
LHCB	AY219853	F: GGCCACTTCTGCAATTCAACA
		R: TACGTCTCATGGTAGCACGG
SGR	EU294209	F: CTCCCTGTGGTTCTGAAGGC
		R: GCTTCTTTGAGTGGACCCCA
β-Tubulin	U91564	F: GCATCTTTGCGTACACTTTGC
		R: ACAACTCGAAACCAACGCTT

F, forward; R, reverse.
